# Letter to the editor regarding "Trends in lumbar epidural injection selection: A survey of practitioner preferences and practice patterns"

**DOI:** 10.1016/j.inpm.2025.100638

**Published:** 2025-08-27

**Authors:** Ryan Triglia, Andrew Walrond, Jesse Wagner, Paul M. Kitei, Jeffrey Boyd, Jeremy I. Simon

**Affiliations:** a Thomas Jefferson University Hospital, Philadelphia, PA, USA; b Rothman Orthopaedic Institute, Philadelphia, PA, USA; c Pennsylvania Pain & Spine Institute, Chalfont, PA, USA

In response to Drs. Aphale et al.,

We are grateful for the thoughtful correspondence from Aphale et al. and their engagement with our recent article [1]. Their insightful commentary is appreciated, and we are emboldened to know that fellow interventional practitioners share our passion for exploring the rationale behind procedure selection in various clinical scenarios. As both interventionalists and educators, we feel a profound responsibility to ground our patient care and procedure choices in robust evidence, and their feedback reinforces the importance of this commitment.

Regarding their first point about the survey’s design, we understand the desire for more comprehensive real-world scenarios. Our intention was to create a survey that respected the time constraints of busy practitioners, as we aimed to avoid responder fatigue and ensure meaningful participation. To achieve this, we crafted idealized scenarios to streamline responses and reduce variables. While this was beyond the scope of our current study, we agree that exploring more complex, real-world scenarios in future studies, would add value.

We also share their concern about the use of particulate steroids as a first-line agent given the potential catastrophic risks [2,3]. It was surprising to observe this trend, especially among interventionalists affiliated with IPSIS. It is also our belief that when particulate steroids are considered for transforaminal epidural steroid injections (ESIs), there should be a well-documented, transparent conversation between the physician and patient, clearly outlining the potential for serious complications. Their emphasis on this point underscores the need for greater awareness and adherence to evidence-based practice.

Their suggestion to incorporate multi-variate statistical analysis to better understand practice patterns is greatly appreciated. We recognize the value this approach could bring in uncovering regional differences and deeper insights. In this initial study with a relatively small sample size, we made the difficult decision to limit the complexity of our statistical analysis, as the data was underpowered in several areas. A future study should be larger and have a more diverse responder pool, which would allow for more robust statistical methods and a clearer picture of procedural trends.

We thank Drs. Aphale et al. again for their suggestions and for taking the time to share their knowledge. We are inspired by their response and look forward to integrating these ideas into future work with a broader group of responders. Their collaboration helps us move closer to our shared goal of advancing evidence-based practice in interventional care.

## Declaration of competing interest

The authors declare that they have no known competing financial interests or personal relationships that could have appeared to influence the work reported in this paper.
